# Dynamics of Telomere Length and Telomerase Activity in the Human Fetal Liver at 5–12 Weeks of Gestation

**DOI:** 10.1155/2018/1385903

**Published:** 2018-07-02

**Authors:** Khrystyna Sorochynska, Nataliia Sych, Alla Duda, Kateryna Kulebyakina, Dmytro Krasnienkov, Alexander Vaiserman, Denys Vatlitsov

**Affiliations:** ^1^Laboratory Department, Cell Therapy Center EmCell, 37A Syretska Str., Kiev 04073, Ukraine; ^2^Clinical Department, Cell Therapy Center EmCell, 37A Syretska Str., Kiev 04073, Ukraine; ^3^Institute of Gerontology, NAMS of Ukraine, 67 Vyshgorodska Str., Kiev 04114, Ukraine

## Abstract

Fetal stem cell- (FSC-) based therapy is a promising treatment option for many diseases. The differentiation potential of FSCs is greater than that in adult stem cells, and they are more tissue-specific and have lower immunogenicity and better intrinsic homing than embryonic ones. Embryonic stem cells have higher proliferative potential than FSCs but can cause teratomas. Therefore, an evaluation of this potential represents an important biomedical challenge. Since regulation of telomere length (TL) is one mechanism governing cellular proliferation, TL is a useful surrogate marker for cell replicative potential. The prenatal dynamics of TL, however, has never been comprehensively studied. In the present study, dynamics of TL and telomerase activity in the human fetal liver during 5–12 weeks of gestation is examined. Both TL and telomerase activity were positively correlated with week of gestation. For both parameters studied, the trend to increase was evident up to 10th week of gestation. After that, they reached a plateau and remained stable. These findings indicate that telomerase activity remains high during the fetal stage, suggesting high replicative capacity of FSCs and their considerable potential for transplantation therapies. These findings, however, are preliminary only due to small sample size and require further evaluation.

## 1. Introduction

Recently, various stem cell-based therapies have received increasing attention regarding their utility in medical applications [1, 2]. All cell types used in this procedure share common functional features such as high self-renewal capability and high potential to generate differentiated cell progenitors of specific lineages both *in vitro* and *in vivo* after transplantation in the host [3]. Due to these properties, stem cells are capable of regenerating different human tissues damaged by disease, injury, or aging. Therefore, the use of stem cells presents a promising strategy in the treatment of many chronic pathological conditions, including cardiac infarction, neurodegenerative disorders, arthritis, diabetes, chronic liver injuries, and neoplastic conditions [4, 5].

Currently, stem cell-based therapies use a variety of embryonic, fetal, and adult stem cells [6]. There are many advantages for the use of fetal stem cells (FSCs) that have led both researchers and clinicians to applying them in regenerative therapy and in treating various disorders [7]. FSCs may be derived from different fetal tissues, including bone marrow, liver, spleen, heart, and brain, after elective termination of pregnancy [8, 9]. These cells exert their therapeutic benefits via activation or inhibition of different molecular and cellular pathways, thereby causing antiapoptotic and anti-inflammatory effects [1]. The intermediate position of FSCs between embryonic and adult stem cells also makes them ideal candidates for reprogramming to a pluripotent state. Their differentiation potential is greater than that in adult stem cells, and they are more tissue-specific than embryonic cells. Since they are isolated from endodermal, ectodermal, and mesodermal lineages, they maintain their tissue-specific cell identities and can therefore be properly regulated, unlike pluripotent embryonic stem cells. FSCs are characterized by lower immunogenicity and better intrinsic homing than embryonic stem cells [9]. They express very low levels of major histocompatibility complex class I and nearly undetectable levels of major histocompatibility complex class II allowing for enhanced transplantation efficiency [10, 11]. Important therapeutic advantage of fetal stem cells as compared to that of early embryonic stem cells is that they do not form teratomas in a host organism [12]. In addition, using these cells is less ethically controversial than embryonic ones [9]. For these reasons, FSC-based therapy is regarded as a promising treatment option for many chronic diseases [4, 5, 13].

The efficiency and duration of therapeutic effects following stem cell transplantation apparently depend on their replicative potential. Therefore, an evaluation of this potential represents an important scientific and clinical challenge. Since the regulation of telomere length (TL) is thought to be one mechanism that governs cellular proliferation, TL has been proposed to be a useful surrogate marker of replicative history and remaining replicative potential of a cell population [14]. Telomeres are repetitive ribonucleoprotein complexes that cap eukaryotic chromosome ends to protect chromosome termini from DNA double-strand breaks [15]. In proliferative cells, the length and integrity of telomeres are maintained by the action of a specialized reverse transcriptase, telomerase [16]. Telomerase activity is generally absent in adult differentiated cells. Therefore, telomeres shorten gradually with each cell division in adult somatic cells (a process known as “telomere attrition”) thereby leading eventually to the arrest of the cell cycle and senescence. In humans, shortened telomeres are regarded as predictors of the risk of age-related cardiovascular disorders commonly associated with retarded cell proliferation and tissue degeneration [17].

Although dynamics of TL is fully characterized from birth to elderly, the prenatal dynamics of TL has never been comprehensively studied to date. It still remains poorly characterized, and available data from few reports are largely contradictory [18]. Main efforts in this research field were focused on investigating preimplantation embryos [19]. TL was found tend to decrease from oocytes to cleavage-stage embryos and tend to increase from cleavage-stage embryos to blastocyst stage embryos [20]. Telomerase activity was found to be high in the male germ line but low or absent in mature oocytes and cleavage stage embryos and then high again in blastocyst stage embryos [21–23]. Data from fetal tissues were recorded only rarely. A pronounced decline in TL throughout 6 and 7 weeks of gestational development was observed in the human fetal tissues in the study by Cheng et al. [24]. After that, TL was slightly shortened until the birth. Telomerase activity assessed as expression of telomerase reverse transcriptase, hTERT, and telomerase RNA component, hTERC, was gradually decreased during 6 to 11 weeks of gestation [24]. In examining the dynamics of TL from 15 to 19 weeks of human gestation, no association between TL and gestational age was found in the study by Youngren et al. [25]. In a longitudinal study conducted in hematopoietic stem cells from the same fetuses across the range of 23–36 weeks of gestational age, TL dynamics was found to be significantly different among fetuses with increasing gestational age [26]. Of the eight fetuses studied, only one showed a significant loss in TL with age, while one demonstrated a significant increase in TL and six showed changes which were below the detection limit at late age points than at early ones. These findings suggest that dynamics of TL during human fetal development is quite complicated and considerable uncertainty exists in this research field. Therefore, further investigation is required to elucidate this point. In the present study, dynamics of TL and telomerase activity during 5–12 weeks of human gestation is examined.

## 2. Materials and Methods

### 2.1. Sampling

21 fetuses of 5–12-week gestational age were used as a source of stem cells. All fetuses were obtained from medical institutions following abortions performed on healthy women due to social indications. Women recruited were preliminarily examined for hemic and viral infections. All procedures for obtaining fetal tissues have been conducted in accordance with current Ukraine ethical and legal standards [27]. The Declaration of Helsinki (2000) and the applicable national standards as they relate to the involvement of human subjects in research were enforced. The study protocol was approved by the Ethics Committees of the Kyiv Institute of Gerontology and Kyiv City Clinical Emergency Hospital. Prior to use, FSC suspensions were stored in the clinic cryobank in liquid nitrogen at −196°C. The parameters for selection of suspensions were as follows: week of gestation (5–12 weeks), counts of nucleated cells (1 to 50 × 10^6^/ml), and also cells viability prior to cryopreservation more than 90%. All FSC suspensions were tested for bacterial and viral infections (HIV-1, HIV-2, HGV, HPV, HBV, HCV, EBV, CMV, HHV6, HSV-1, 2, *Treponema pallidum*, rubella, parvovirus B19, *Mycoplasma hominis*, *Mycoplasma genitalium*, *Toxoplasma gondii*, *Chlamydia trachomatis*, *Ureaplasma parvum*, and *Urealyticum*). Suspension defrosting was performed in accordance with standard protocols.

### 2.2. TL Measurement

The relative telomere lengths (RTLs) were measured by a multiplex real-time quantitative polymerase chain reaction (qPCR) [28]. DNA was extracted from the fetal liver cell suspension using a standard protocol for phenol-chloroform DNA extraction [29]. PCR reaction mix was prepared using a commercial reagent kit Luna® Universal qPCR and RT-qPCR (New England Biolabs) with the addition of betaine (Sigma-Aldrich) at a final concentration of 1 M. For multiplex qPCR, the telomere primer pair telg and telc (final concentrations 450 nM each) were combined with the albumin primer pair albu and albd (final concentrations 250 nM each) in the master mix. The list of primers used for RT-qPCR analysis is given in Table 1 below. The thermal cycling profile was as follows: 15 min at 95°C, 2 cycles of 15 s at 94°C, 15 s at 49°C and 32 cycles of 15 s at 94°C, 10 s at 62°C, 15 s at 74°C with signal acquisition, 10 s at 84°C, and 15 s at 88°C with signal acquisition. To obtain the calibration curve, PCR was carried out at four concentrations of the reference DNA in duplicate which cover a range of 27-fold dilutions prepared by a serial dilution. All DNA samples were run in triplicates. Amplification curves were generated by the Opticon Monitor 3 software. For this purpose, after thermal cycling and raw data collection were complete, the Opticon Monitor 3 software was used to generate two standard curves for each plate, one for the telomere signal and another for the single-copy gene (scg) albumin signal. RTLs were expressed as a *T*/*S* ratio, that is, the telomere repeat copy number (*T*) to the scg copy number (*S*).

### 2.3. Telomerase Activity Measurements

Telomerase activity was assessed by real-time qPCR [28]. Fetal liver cells and HEK293 (positive control) were lysed in Invitrogen's NP-40 lysis buffer (50 mM Tris, pH 7.4, 250 mM NaCl, 5 mM EDTA, 50 mM NaF, 1 mM Na_3_VO_4_, 1% Nonidet™ P40 (NP40) 0.02% NaN_3_) with 1 mM PMSF (Sigma-Aldrich) and 10 *μ*l/ml (*v*/*v*) Protease Inhibitor Cocktail (Sigma-Aldrich) on ice. Subsequent centrifugation was performed at 16400*g* for 20 min at +4°C. 180 *μ*l of supernatant was transferred to a fresh tube, and protein concentration measurements were determined using Pierce™ BCA Protein Assay Kit (Thermo Scientific) according to the manufacturer's protocols.

The reaction mixture for TRAP was prepared on the basis of Luna Universal qPCR and RT-qPCR (New England Biolabs) with 5 mM EGTA, 4 ng/ml ACX, and 4 ng/ml oligo TS. 2 *μ*l of lysate was added to 23 *μ*l of TRAP mix and was incubated for 30 min at 30°C. Then, the PCR was performed at following conditions: 95°C 1 min; 40 cycles of 95°C for 15 s, and 60°C for 1 min with signal acquisition. PCR products were quantified with a Chromo4 (Bio-Rad) and analyzed with Opticon Monitor v3.1 software. HEK293 cells were used for standard curve generation. For this purpose, five 5-fold dilutions were prepared.

### 2.4. Statistical Analysis

The Kolmogorov-Smirnov test was used to assess normality of distribution of variables. Parametric tests such as linear regression and Pearson correlation were performed to evaluate the association between variables. To assess a trend across ordered categorical variables, a one-way ANOVA followed by Tukey's HSD post hoc tests was applied for pairwise comparisons among different two-week age groups and univariate test of significance for planned comparison was applied to assess a common trend across all age groups. All analyses were performed by Statistica 8.0 software (StatSoft Inc., USA).

## 3. Results

**Figure 1:**
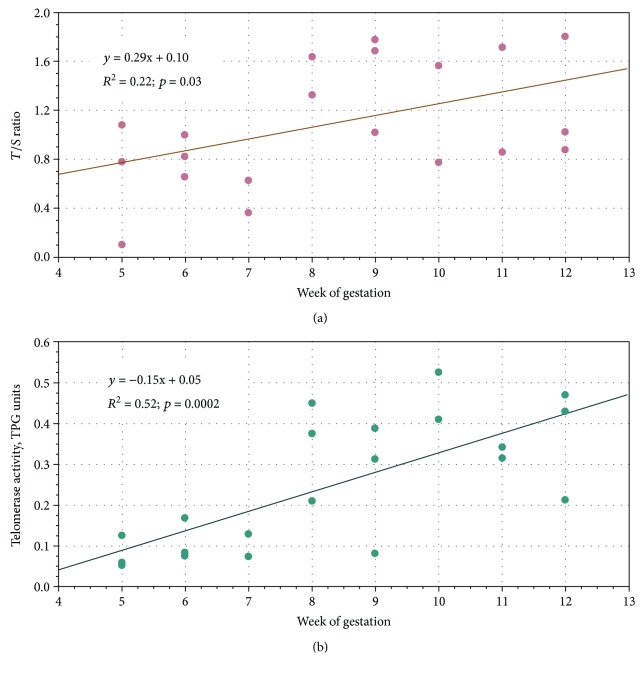
Regression plots of TL (a) and telomerase activity (b) in human fetal livers against week of gestation.

No significant deviation from normal distribution was observed (for both parameters studied, *p* > 0.05 by Kolmogorov-Smirnov test). Therefore, statistical analyses were performed by parametric tests. Age trend significance was evaluated by a linear regression model. As we can see from Figure 1, both TL (*T*/*S* ratio) and telomerase activity were positively correlated with week of gestation (*T*/*S* ratio, Pearson correlation *r* = 0.47, *p* = 0.03; telomerase activity, *r* = 0.72, *p* = 0.0002).

**Figure 2:**
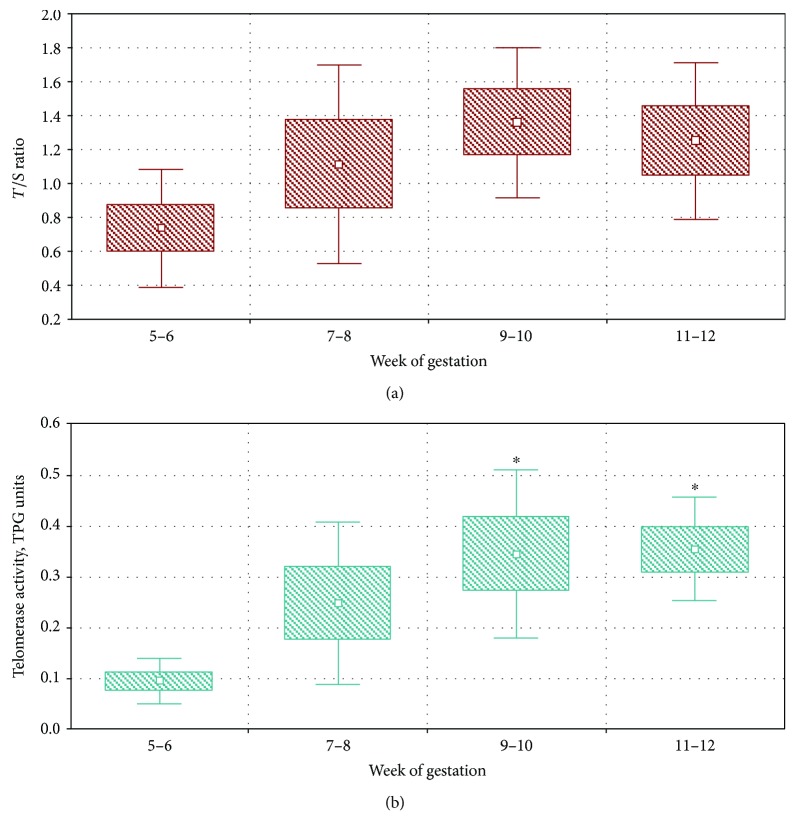
Box-and-whisker plots showing the values of TL (a) and telomerase activity (b) in human fetal livers in different two-week periods of gestation. In each box-and-whisker plot, the box represents the standard error, the white square inside the box represents the mean value, and the whiskers above and below the box indicate the standard deviation. The asterisks indicate significant differences from the 5-6-week age group by Tukey's HSD post hoc tests.

**Figure 3:**
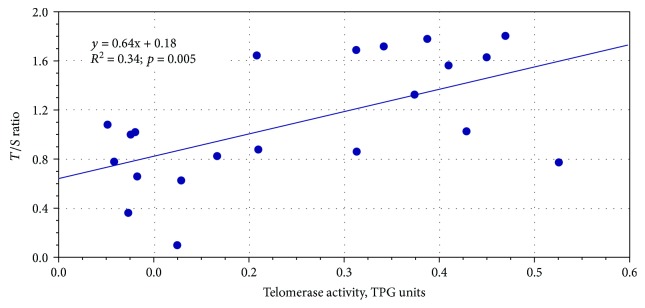
Regression plot of TL against telomerase activity levels.

The trend to increase of TL and telomerase activity with gestational age was also evident from Figure 2, where box-and-whisker plots for two-week periods of gestation are shown. This trend was highly significant for telomerase activity (univariate test of significance for planned comparison, *F* = 13.07, *p* = 0.002), and it was borderline significant for TL (*F* = 4.07, *p* = 0.06). The trend to increase was evident at least up to the 10th week of gestation for both parameters studied; after that, they reached a plateau and remained stable. Moreover, as expected, a significant positive correlation (*r* = 0.59, *p* = 0.005) was observed between TL and telomerase activity levels (Figure 3).

## 4. Discussion

The human fetal liver is considered as a valuable cell source for cell transplantation therapy [30]. Numerous stem cell compartments composed of various stem or progenitor cells and related cell lineages have been revealed by anatomical examination of fetal livers [31]. There are, in particular, hepatic stem and progenitor cells inside the ductal plates and also multipotent stem and progenitor cells inside the extrahepatic bile ducts and large intrahepatic bile ducts [32–35]. Both these niches are accompanied by mesenchymal companion cells. In addition, the hepatic parenchyma contains a lot of other mesenchymal cells including hematopoietic and endothelial stem cells as well as macrophages [33]. Since hematopoiesis is the main function of the fetal liver during 5 to 12 weeks of gestation, the concentration of hematopoietic stem cells is higher than that of any other cell type and hepatocyte proportion increases with gestational age at this developmental stage. All of the abovementioned cell types may be used as attractive sources for cell transplantation in regenerative medicine and in disease therapies [35].

Until now, the question remains unresolved about the replicative potential of these cell lineages. It is commonly believed that telomerase is activated and maintains TL in embryonic stem cells and after the blastocyst stage, the level of telomerase activity is low or absent in the majority of stem cells [36]. Therefore, it is assumed that replicative potential of FSCs is limited by progressive telomere shortening during fetal development. Our findings, however, demonstrate that telomerase activity may remain high throughout this stage. In particular, our results strongly contradict the findings of Cheng et al. [24] who found a pronounced decline in both TL and telomerase activity throughout 6 to 11 weeks of human gestation. This contradiction can be probably explained by the fact that there are organ-specific differences in the rate of telomere attrition during fetal life, with a higher rate of telomere shortening prenatally in some organs and postnatally in others [37]. Indeed, unlike Cheng and coauthors who analyzed mixed fetal tissues, we investigated the fetal liver tissue only. The telomerase activity may apparently persist in this tissue through the 12th gestational week since hepatic cell precursors begin to differentiate into fetal liver cells (hepatoblasts), and moreover, highly proliferative hematopoietic stem cells are widely represented in the human fetal liver at this stage of development [38]. Increase of TL and telomerase activity with an increase in gestational age indicates that the proliferative potential of FSCs may even increase with the increasing term of gestation, at least during the period studied. Thus, the fetal liver can likely be a source of a large pool of hematopoietic and other stem cells and would therefore be highly useful in transplantation therapies.

Finally, we would like to emphasize strengths and limitations of the present study. Unlike previous studies on the topic, where qualitative or semiquantitative methods were applied to measure the TL and telomerase activity, we used the highly sensitive and reproducible quantitative methods in the present research. Moreover, our findings seem quite innovative especially given the fact that we used fetal liver stem cells only in our research. This seems a very important point because mixed fetal tissues were used in most previous studies, complicating conclusions regarding causal inference. The main limitation of our study is that the sample size is too small to be generalized to the whole population and to draw definitive conclusions. Therefore, our findings should be considered as preliminary only and need to be further evaluated in larger samples.

## Figures and Tables

**Table 1:** List of primers used for RT-qPCR quantification.

Primer name	Primer sequence
TS	5′-AATCCGTCGAGCAGAGTT-3′
ACX	5′-GCGCGGCTTACCCTTACCCTTACCCTAACC-3′
telg	5′-ACACTAAGGTTTGGGTTTGGGTTTG GGTTTGGGTTAGTGT-3′
telc	5′-TGTTAGGTATCC CTATCCCTATCCCTATCCCTATCCCTAACA-3′
albu	5′-CGGCGGCGGGCGGCGCGGGCTGGGCGGAA ATGCTGCACAGAATCCTTG-3′
albd	5′-GCCCGGCCCGCCGCG CCCGTCCCGCCGGAAAAGCATGGTCGCCTGTT-3′

## Data Availability

The data used to support the findings of this study are available from the corresponding author upon request.
